# Supplementary material for: Identification and Characterization of a Novel Species of Genus Akkermansia with Metabolic Health Effects in a Diet-Induced Obesity Mouse Model
Source: Cells. 2022 Jun 30;11(13):2084. doi: 10.3390/cells11132084 (PMC9265676; doi:10.3390/cells11132084)
Supplement: Supplementary file 1 [file cells-11-02084-s001.zip › cells-1765106-supplementary.pdf]

cells

MDPI

*Article*

# Identification and Characterization of a Novel Species of Genus *Akkermansia* with Metabolic Health Effects in a Diet-Induced Obesity Mouse Model

**Ritesh Kumar [1,*], Helene Kane [1], Qiong Wang [1], Ashley Hibberd [2], Henrik Max Jensen [3], Hye-Sook Kim [1], Steffen Yde Bak [3], Isabelle Auzanneau [4], Stéphanie Bry [4], Niels Christensen [3], Andrew Friedman [1], Pia Rasinkangas [5], Arthur C. Ouwehand [5], Sofia D. Forssten [5] and Oliver Hasselwander [6]**

[1] Health & Biosciences, International Flavors & Fragrances, Inc. (1FF), Wilmington, DE 19803, USA; helene-m.a.kane@iff.com (H.K.); qiong.wang@iff.com (Q.W.); hskim1214@gmail.com (H.-S.K.); andrew.friedman@iff.com (A.F.)
[2] Health & Biosciences, IFF, Saint Louis, MO 63110, USA; ashley.hibberd@iff.com (A.H.)
[3] Health & Biosciences, IFF, 8220 Brabrand, Denmark; henrik.max.jensen@iff.com (H.M.J.); steffen.yde.bak@iff.com (S.Y.B.); niels.christensen@iff.com (N.C.)
[4] Health & Biosciences, IFF, 86270 Dange, France; isabelle.auzanneau@iff.com (I.A.); stephanie.bry@iff.com (S.B.)
[5] Health & Biosciences, IFF, 02460 Kantvik, Finland; pia.rasinkangas@iff.com (P.R.); arthur.ouwehand@iff.com (A.C.O.); sofia.forssten@iff.com (S.D.F.)
[6] Health & Biosciences, IFF, c/o Danisco UK Ltd., Reigate United RH2 9PW, UK; oliver.hasselwander@iff.com
* Correspondence: ritesh.kumar@iff.com; Tel.: +1-302-379-4738

## Supplementary Information

**Figure S1: The protein sequences from 235 public *Akkermansia* genomes and DSM 33459 genome were clustered at 95% aa identity. The distance between each pair of genomes was estimated using the percent of clusters not shared between them. The clusters by hierarchical clustering were visualized in a dendrogram and a heatmap. The closer the genomes are, the smaller the distance on the dendrogram.**

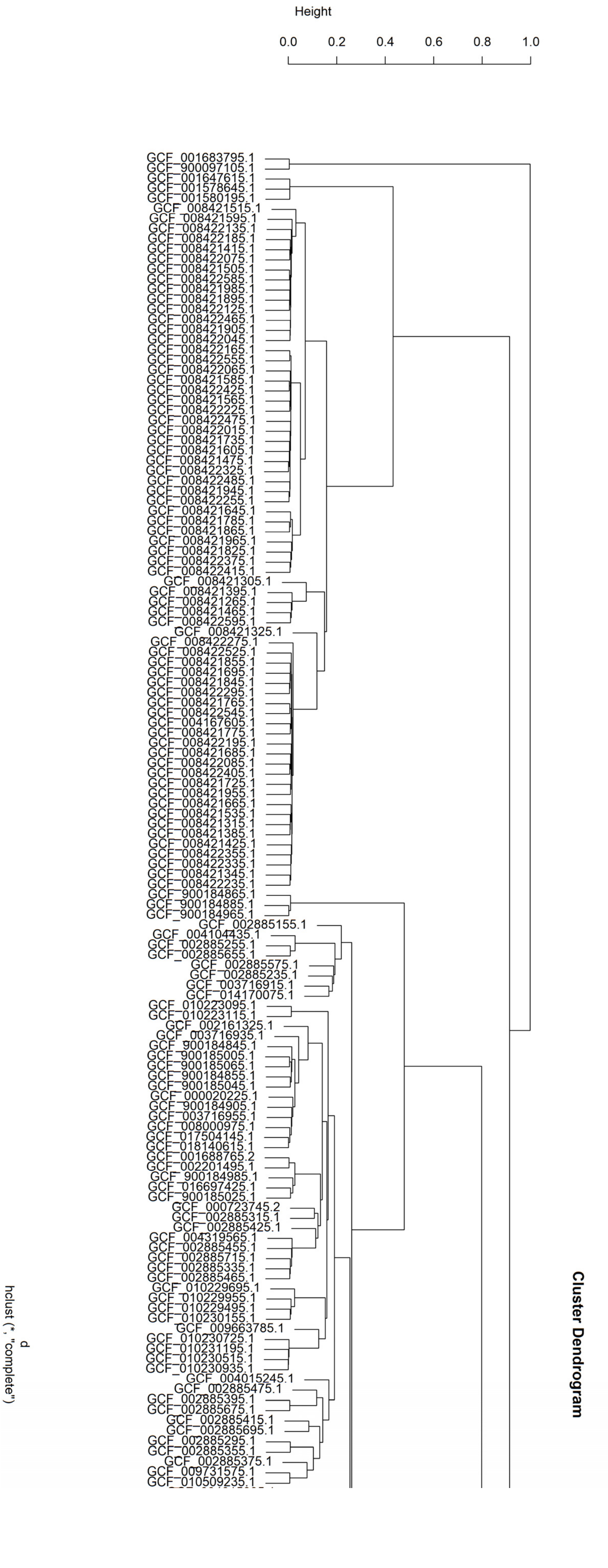
Cluster Dendrogram
Height
0.0 0.2 0.4 0.6 0.8 1.0
d
hclust (*, "complete")

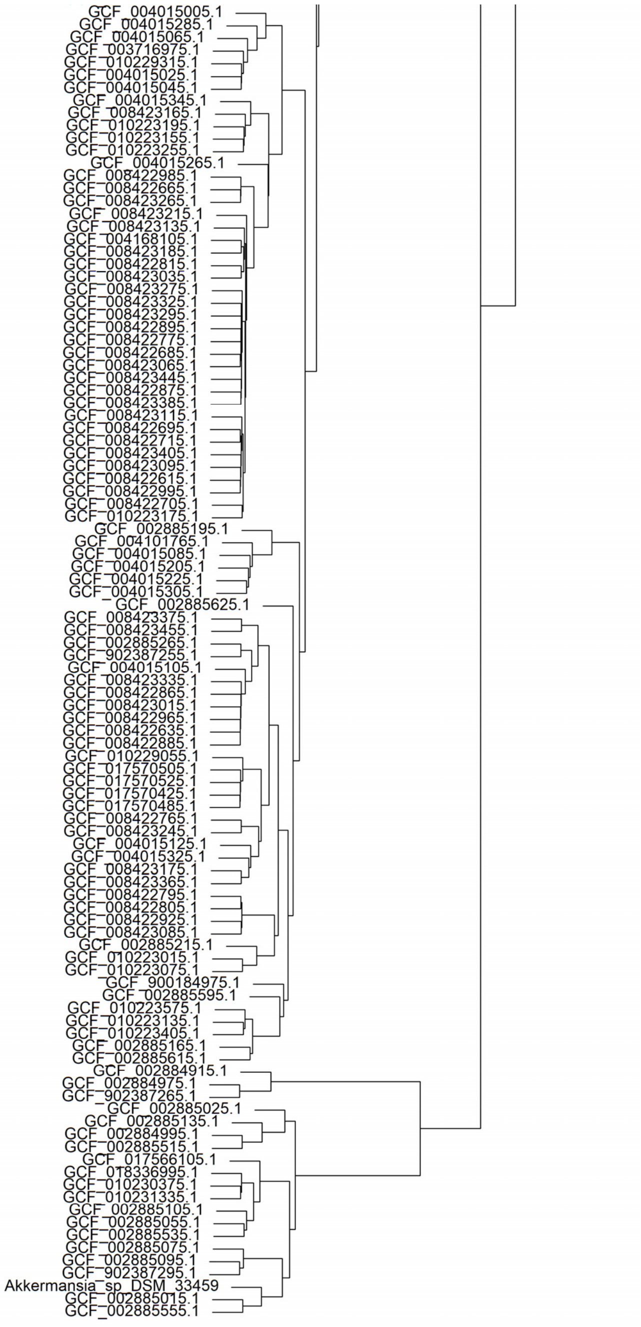
GCF_004015005.1
GCF_004015285.1
GCF_004015065.1
GCF_003716975.1
GCF_010229315.1
GCF_004015025.1
GCF_004015045.1
GCF_004015345.1
GCF_008423165.1
GCF_010223195.1
GCF_010223155.1
GCF_010223255.1
GCF_004015265.1
GCF_008422985.1
GCF_008422665.1
GCF_008423265.1
GCF_008423215.1
GCF_008423135.1
GCF_004168105.1
GCF_008423185.1
GCF_008422815.1
GCF_008423035.1
GCF_008423275.1
GCF_008423325.1
GCF_008423295.1
GCF_008422895.1
GCF_008422775.1
GCF_008422685.1
GCF_008423065.1
GCF_008423445.1
GCF_008422875.1
GCF_008423385.1
GCF_008423115.1
GCF_008422695.1
GCF_008422715.1
GCF_008423405.1
GCF_008423095.1
GCF_008422615.1
GCF_008422995.1
GCF_008422705.1
GCF_010223175.1
GCF_002885195.1
GCF_004101765.1
GCF_004015085.1
GCF_004015205.1
GCF_004015225.1
GCF_004015305.1
GCF_002885625.1
GCF_008423375.1
GCF_008423455.1
GCF_002885265.1
GCF_902387255.1
GCF_004015105.1
GCF_008423335.1
GCF_008422865.1
GCF_008423015.1
GCF_008422965.1
GCF_008422635.1
GCF_008422885.1
GCF_010229055.1
GCF_017570505.1
GCF_017570525.1
GCF_017570425.1
GCF_017570485.1
GCF_008422765.1
GCF_008423245.1
GCF_004015125.1
GCF_004015325.1
GCF_008423175.1
GCF_008423365.1
GCF_008422795.1
GCF_008422805.1
GCF_008422925.1
GCF_008423085.1
GCF_002885215.1
GCF_010223015.1
GCF_010223075.1
GCF_900184975.1
GCF_002885595.1
GCF_010223575.1
GCF_010223135.1
GCF_010223405.1
GCF_002885165.1
GCF_002885615.1
GCF_002884915.1
GCF_002884975.1
GCF_902387265.1
GCF_002885025.1
GCF_002885135.1
GCF_002884995.1
GCF_002885515.1
GCF_017566105.1
GCF_018336995.1
GCF_010230375.1
GCF_010231335.1
GCF_002885105.1
GCF_002885055.1
GCF_002885535.1
GCF_002885075.1
GCF_002885095.1
GCF_902387295.1
Akkermansia_sp_DSM_33459
GCF_002885015.1
GCF_002885555.1

**Table S1**. Susceptibility of the quality control strain *Bacteroides fragilis* DSM 2151 (ATCC 25285) to selected antimicrobials using Brucella broth (standard medium). The MIC Quality Control ranges for anaerobes, specifically for *B. fragilis* DSM 2151 (ATCC 25285), are shown in the right-hand column (CLSI standard M100, 31st ed., Table 5E) [68].

| Antimicrobial | MIC (µg/mL) | *B. fragilis* DSM 2151 (ATCC 25285) (µg/mL) |
|---|---|---|
| Ampicillin | 16 | -[a] |
| Ampicillin-sulbactam | 1/0.5 | 0.5/0.25-2/1 |
| Benzylpenicillin | >8 | -[a] |
| Penicillin | 8 | 8-32 |
| Gentamicin | >256 | -[a] |
| Kanamycin | >512 | -[a] |
| Streptomycin | 512 | -[a] |
| Clindamycin | 1 | 0.5-2 |
| Tetracycline | ≤0.25 | -[a] |
| Ciprofloxacin | 2 | -[a] |
| Colistin | >8 | -[a] |
| Fosfomycin* | >128 | -[a] |
| Vancomycin | 64 | -[a] |
| Erythromycin | 4 | -[a] |
| Chloramphenicol | 4 | 4-16 |
| Imipenem | 0.12 | 0.03-0.25 |
| Meropenem | 0.06 | 0.03-0.25 |
| Metronidazole | 0.5 | 0.25-2 |
| Trimethoprim | 8 | -[a] |

[a] No MIC values for ampicillin, benzylpenicillin, gentamicin, kanamycin, streptomycin, tetracycline, ciprofloxacin, colistin, fosfomycin vancomycin, erythromycin or trimethoprim are available against the quality control strain *Bacteroides fragilis* DSM2151 (ATCC 25285).

*Reinforced medium for Clostridia agar were used

**Table S2**. Susceptibility of the quality control strain *Bacteroides fragilis* DSM 2151 (ATCC 25285) to selected antimicrobials using YCFAC broth (growth medium for *Akkermansia sp.* DSM 33459). The MIC Quality Control ranges for anaerobes, specifically for *B. fragilis* DSM 2151 (ATCC 25285), are shown in the right-hand column (CLSI standard M100, 31st ed., Table 5E) [68].

| Antimicrobial | MIC (µg/mL) | *B. fragilis* DSM 2151 (ATCC 25285) (µg/mL) |
|---|---|---|
| Ampicillin | >32 | -[a] |
| Ampicillin-sulbactam | 2/1 | 0.5/0.25-2/1 |
| Benzylpenicillin | >8 | -[a] |
| Penicillin | >16 | 8-32 |
| Gentamicin | >256 | -[a] |
| Kanamycin | >512 | -[a] |
| Streptomycin | >512 | -[a] |
| Clindamycin | 0.5 | 0.5-2 |
| Tetracycline | 0.5 | -[a] |
| Ciprofloxacin | 4 | -[a] |
| Colistin | >8 | -[a] |
| Fosfomycin* | >128 | -[a] |
| Vancomycin | 64 | -[a] |
| Erythromycin | 4 | -[a] |
| Chloramphenicol | 4 | 4-16 |
| Imipenem | >32 | 0.03-0.25 |
| Meropenem | >32 | 0.03-0.25 |
| Metronidazole | 2 | 0.25-2 |
| Trimethoprim | >64 | -[a] |

[a] No MIC values for ampicillin, benzylpenicillin, gentamicin, kanamycin, streptomycin, tetracycline, ciprofloxacin, colistin, fosfomycin vancomycin, erythromycin or trimethoprim are available against the quality control strain *Bacteroides fragilis* DSM2151 (ATCC 25285).

*Reinforced medium for Clostridia agar were used

**Table S3**: Genome comparison between *Akkermansia* sp. DSM 33459 and *A. muciniphila* ATCC BAA-835 or the presence of Corrin ring biosynthesis gene cluster.

| Gene | *Akkermansia sp.* DSM 33459 | *A. muciniphilia* ATCC BAA-835 | ECnumber | product |
|---|---|---|---|---|
| **cbiA** | 1 | 0 | 6.3.5.11 | Cobyrinate a,c-diamide synthase |
| **cbiC** | 1 | 0 | 5.4.99.60 | Cobalt-precorrin-8 methylmutase |
| **cbiD** | 1 | 0 | 2.1.1.195 | Cobalt-precorrin-5B C(1)-methyltransferase |
| **cbiET** | 1 | 0 | unknownEC | Cobalamin biosynthesis bifunctional protein CbiET |
| **cbiF** | 1 | 0 | 2.1.1.271 | Cobalt-precorrin-4 C(11)-methyltransferase |
| **cbiKp** | 1 | 0 | 4.99.1.3 | Sirohydrochlorin cobaltochelatase CbiKP |
| **cbiL** | 1 | 0 | 2.1.1.151 | Cobalt-precorrin-2 C(20)-methyltransferase |
| **chiA1** | 1 | 0 | 3.2.1.14 | Chitinase A1 |
| **cobO** | 1 | 0 | 2.5.1.17 | Cob(I)yrinic acid a,c-diamide adenosyltransferase |

**Table S4**: Global Liver proteomics analysis: Significant proteins are distinctly regulated in DIO mice in Akk$^{Gly}$ and Liraglutide groups.

| Peroxisomal proteins | Gene | AkkGly* | Liraglutide* | AkkGly/Vehicle# | Liraglutide/Vehicle# |
|---|---|---|---|---|---|
| Peroxisomal bifunctional enzyme | Ehhadh | -2.29 | -4.89 | 1.17 | 1.35 |
| Peroxisomal coenzyme A diphosphatase NUDT7 | Nudt7 | -2.43 | -3.39 | 1.26 | 1.29 |
| Peroxisomal membrane protein 4 | Pxmp4 | 0.49 | -3.38 | 0.86 | 1.98 |
| Peroxisome assembly factor 2 | Pex6 | -3.56 | -3.34 | 1.67 | 1.57 |
| 3-ketoacyl-CoA thiolase A, peroxisomal | Acaa1a | -0.87 | -3.34 | 1.06 | 1.35 |
| Peroxisomal targeting signal 1 receptor | Pex5 | -2.3 | -3.29 | 1.21 | 1.36 |
| Peroxisomal carnitine O-octanoyltransferase | Crot | -0.2 | -2.9 | 0.94 | 1.32 |
| Glutathione peroxidase 1 | Gpx1 | 0.1 | -2.9 | 1.06 | 1.26 |
| Peroxisomal membrane protein 2 | Pxmp2 | -2.62 | -2.54 | 1.19 | 1.20 |
| Copper chaperone for superoxide dismutase | Ccs | -0.55 | -2.52 | 1.03 | 1.23 |
| Peroxisomal membrane protein 11A | Pex11a | -2.19 | -1.95 | 1.20 | 1.15 |
| Peroxiredoxin-5, mitochondrial | Prdx5 | -2.45 | -0.8 | 1.15 | 1.07 |
| Peroxisomal membrane protein PEX14 | Pex14 | -0.28 | 2.00 | 0.97 | 0.71 |
| Peroxisomal multifunctional enzyme type 2 | Hsd17b4 | 0.33 | 2.12 | 0.96 | 0.85 |
| 3-ketoacyl-CoA thiolase B, peroxisomal | Acaa1b | 1.91 | 2.12 | 0.81 | 0.72 |
| Peroxisomal sarcosine oxidase | Pipox | 1.22 | 2.45 | 0.97 | 0.92 |
| Glutathione peroxidase 3 | Gpx3 | 0.51 | 2.61 | 0.96 | 0.73 |
| Peroxisomal trans-2-enoyl-CoA reductase | Pecr | -0.48 | 2.74 | 1.02 | 0.88 |
| Peroxiredoxin-2 | Prdx2 | -0.38 | 2.74 | 1.01 | 0.88 |
| Peroxisomal 2,4-dienoyl-CoA reductase | Decr2 | 0.50 | 3.02 | 0.82 | 0.66 |
| Thioredoxin-dependent peroxide reductase, mitochondrial | Prdx3 | -0.04 | 3.22 | 0.98 | 0.75 |
| Peroxisomal acyl-coenzyme A oxidase 1 | Acox1 | 1.90 | 3.64 | 0.85 | 0.75 |
| Peroxisomal membrane protein PEX16 | Pex16 | 1.41 | 3.66 | 0.92 | 0.79 |
| Peroxisomal biogenesis factor 19 | Pex19 | 3.18 | 3.77 | 0.48 | 0.14 |
| Peroxisomal membrane protein PMP34 | Slc25a17 | 1.61 | 3.86 | 0.85 | 0.56 |
| Peroxisome biogenesis factor 1 | Pex1 | -1.64 | 3.91 | 1.03 | 0.87 |
| Peroxisomal biogenesis factor 3 | Pex3 | 1.15 | 6.12 | 0.89 | 0.58 |
| Peroxisomal membrane protein PEX13 | Pex13 | 0.72 | 6.28 | 0.77 | 0.14 |
| Peroxisomal membrane protein 11C | Pex11g | 1.12 | 7.95 | 0.95 | 0.66 |
| | | | *Z-scores | | #Fold |
